# Differential gene expression profile of retinoblastoma compared to normal retina

**Published:** 2010-07-13

**Authors:** Arupa Ganguly, Carol L. Shields

**Affiliations:** 1 Department of Genetics, University of Pennsylvania, School of Medicine, Philadelphia, PA; 2 Ocular Oncology Services, Wills Eye Institute, Thomas Jefferson University, Philadelphia, PA

## Abstract

**Purpose:**

The retinoblastoma gene (*RB1*) is a tumor suppressor gene that was first discovered in a rare ocular pediatric tumor called retinoblastoma (RB). The *RB1* gene is essential for normal progression through the cell cycle and exerts part of its function through the family of transcription factors (E2F) and many other intermediaries. In the absence of normal *RB1*, genomic instability and chromosomal aberrations accumulate, leading to tumor initiation, progression, and ultimately metastasis. The purpose of this report was to identify the molecular pathways that are deregulated in retinoblastoma.

**Methods:**

We compared gene expression signatures of matched normal retinal tissue and retinoblastoma (RB) tumor tissue from six individuals, using microarray analysis followed by statistical and bioinformatic analyses.

**Results:**

We identified 1,116 genes with increased expression and 837 with decreased expression in RB tumor tissue compared to matched normal retinal tissue. Functional categories of the cognate genes with the greatest statistical support were cell cycle (309 genes), cell death (437 genes), DNA replication, recombination and repair (270 genes), cellular growth and proliferation (464 genes), and cellular assembly and organization (110 genes). The list included differentially expressed retinal cone-cell-specific markers. These data indicated the predominance of cone cells in RB and support the idea that the latter group of cells may be the cells of origin for RB.

**Conclusions:**

The genes differentially expressed in RB as compared to normal retina belong mainly to DNA damage-response pathways, including, but not limited to, breast cancer associated genes *(BRCA1, BRCA2),* ataxia telangiectasia mutated gene *(ATM),* ataxia telangiectasia *and Rad3* related gene*(ATR), E2F,* checkpoint kinase 1 *(CHK1)* genes. In addition, novel pathways, such as aryl hydrocarbon receptor (AHR) signaling, polo-like kinase and mitosis, purine metabolism pathways were involved. The molecules AHR, CHK1, and polo-like kinases are of particular interest because there are several currently available drugs that target these molecules. Further studies are needed to determine if targeting these pathways in RB will have therapeutic value. It is also important to evaluate the relative importance of these pathways in different cells that make up the normal retina.

## Introduction

Retinoblastoma (RB) is a rare pediatric ocular tumor arising from immature neuroectodermal cells of the retina. Mutations and/or epigenetic alterations inactivating both alleles of the retinoblastoma gene (*RB1*) are associated with RB. The mean age-adjusted incidence of RB in the USA is 11.8 per million children aged 0–4 years, and there is no significant variation in incidence between genders or among races [1]. The incidence of bilateral disease is approximately 26.7% versus 71.9% of unilateral cases [1]. Germline mutations on one allele of *RB1* are associated with a younger age of onset and bilateral disease. Second malignancies can occur at the site of radiation or at distant sites, including bone (osteosarcoma), skin (melanoma), brain, bladder, and lung [2–4]. Approximately 90% of children carrying a germline mutation in *RB1* will develop retinoblastoma during their early childhood [5–8].

Inactivation of the retinoblastoma protein (pRB) promotes genomic instability and results in chromosomal deletions, duplications, and rearrangements [9–11]. pRB is a nuclear protein that is critical for cell cycle exit and terminal differentiation of retinal cells. When hypo-phosphorylated, pRB combines with members of the transcription factor (E2F) family of transcription factors that regulate cell-cycle progression. The expression levels of different E2F target genes reflect the positive or negative control exerted by pRB binding in different signaling pathways. Although pRB was discovered through its role as a tumor suppressor, it is also involved in tumor progression and metastasis. The role of pRB is widespread and complex, as indicated by the fact that it binds to over 100 different proteins and has many transcription targets [11].

The gene expression signature of single RB tumors has been examined to a limited extent. In a study of primary tumors with gains of chromosome 1q, 24 genes on several chromosomes had significantly increased expression compared with tumors that did not have chromosome 1q gains [12]. In a second study, comparison of gene expression in ten retinoblastoma tumors with three normal retinal samples collected from unmatched adult donors indicated that 481 genes were downregulated and over 1,000 genes were upregulated [13,14].

Knowledge of the molecular pathways controlled by pRB is essential for understanding normal growth and development, tumor suppression, and tumor progression. Identification of these pathways carries the potential for development of effective therapeutic targets. To identify the critical molecular pathways that are affected by the absence of functional pRB, we compared gene expression patterns in RB and matched normal retinal tissue from six individuals.

## Methods

### Retinoblastoma tumor and normal retinal specimens

Fresh retinoblastoma tissue was collected following enucleation in the Oncology Service at the Wills Eye Institute (Table 1), Thomas Jefferson University (C.L.S.), Philadelphia, PA, and sent to the Genetics Diagnostic Laboratory (A.G.), University of Pennsylvania, Philadelphia, for genetic testing. The informed consent is received before surgery. As the patients are all under the age of five, the parent(s) or legal guardians sign the consent form. The technique of tissue harvesting has been described in the literature [15]. In brief, the tumor tissue was harvested following enucleation on a separate tray by the operating surgeon (C.L.S.). The globe was opened with an 8.0-mm corneal trephine (Medtronics, Jacksonville, FL) so that the trephine straddled a margin of the tumor within the eye. The sclera was carefully opened so that seeding was avoided, and the choroidal tissue was incised. The retinoblastoma tumor was visualized, and tissue was obtained using a 6-mm tissue spoon and Wescott scissors. After harvesting of retinoblastoma from the enucleated globe, the tissue was inspected and if the tumor was well circumscribed and without tumor seeding, a sample of normal uninvolved retina in an opposite quadrant was harvested and submitted separately to avoid contamination from the tumor site. The degree of contamination is expected to be minimal as judged by visual inspection. The specimen was flash frozen on dry ice and transferred for genetic analysis. The globe and scleral cap were placed in formalin and sent to the pathology laboratory. The protocol for genetic analysis of RB tumors was approved by the Institutional Review Board of the University of Pennsylvania (protocol number 706577, originally approved March 2005).

**Table 1:** Clinical feature of the individuals with retinoblastoma and the tumors.

**Patient description**		RB1 mutation*	**Laterality**	**Differentiation**	**Clinical features**					
**Age**	**Gender**	Retino-blastoma Stage ICRB*	**Growth**	**Optic nerve invasion**	**Iris neo-vascularization**	**Retinal detachment**	**Necrosis (%)**
13 months	Female	p.E125X, exon 3; germline	Bilateral	well, poor	E					40
9 months	Female	g.73774G>T, exon 13, E413X; germline	Bilateral	well, variable	E	Endo/exo-phytic	No	Not reported	Yes	20
15 months	Female	g.76490A>G, +4 position of intron 14; LOH; somatic	Unilateral	poor	E	Endophytic	No	Yes	Yes	10
5 years	Male	g.153358G>A, +5 position of intron 19; LOH;somatic	Unilateral	poor	D	Endophytic	No	No	Yes	20
4 years	Female	RB1 gene duplication;	Unilateral	poor	E	Endo/exo-phytic	No	Not reported	Yes	80
12 months	Female	Promoter methylation; somatic	Unilateral	well, variable	E	Endo/exo-phytic	No	No	Yes	50

* ICRB - International Classification of Retinoblastoma 18]. There was no evidence of uveal invasion in any RB tumor.

### Isolation of DNA

Genomic DNA was isolated from normal retina and frozen tumors using a commercial DNA isolation kit (Gentra, Minneapolis, MN), following the manufacturer’s instructions including proteinase-K digestion followed by salting out of DNA.

### Mutation analysis of coding sequences of *RB1* and *TP53*

Mutation analysis was performed on DNA isolated from normal retina and RB samples to identify mutations in the 27 coding exons of the *RB1* gene and the ten coding exons of the tumor protein 53 (*TP53)* gene. The primers and PCR conditions for sequencing are included in Table 2. In addition to scanning the coding sequences, analyses for detection of mutations involving gene deletion, duplication, or rearrangements within the gene and methylation of regulatory regions of the *RB1* and *TP53* genes in tumor DNA samples were performed.

**Table 2:** Primer Sequences for all coding exons of RB1 gene.

**Exon**	**Forward primer (5′-3′)**	**Reverse primer (5′-3′)**
1	GTTTTTCTCAGGGGACGTTGAA	CCAGAATCCTGTCACCATTCT
1	ACGTGCGCGCGCGTCGT	CCGGCCCCTGGCGAGGA
2	TATTTTGGAATGACCATGAAAAAGA	AGAGGTAAATTTCCTCTGGGTAAT
3	ATTAGTGTGAAATGAAATCCTTTCA	CCAGGACACAAACTGCTACCT
4	TAGTGATTTGATGTAGAGCTGATA	GCATTCAGAATGCATATTACTGGA
5	TGGGAAAATCTACTTGAACTTTGT	CTTCTTTGTAGTACAAGGCATGTA
6	GCATTCTATTATGCATTTAACTAAG	GTTAATAAGCCAAGCAGAGAATGA
7	GGATATACTCTACCCTGCGATTT	CTGTCAGCCTTAGAACCATGTT
8	CCTAAGTTATAGTTAGAATACTTCAT	AAACATGCTCATAACAAAAGAAGTA
9	CTTACCCTGCATTGTTCAAGAGT	CAGTAAATTGATCTAAGAAAGTTAGA
10	ATATTGCATGCGAACTCAGTGTAT	TGATATCTAAAGGTCACTAAGCTAA
11	GATTTTATGAGACAACAGAAGCATT	TCCACCACACCTGGCCTTCAA
12	AAACCACAGTCTTATTTGAGGGAA	ATAACTACATGTTAGATAGGAGATT
13	AAAAAGTCATATATTATGGAGCAGAA	CGAACTGGAAAGATGCTGCTT
14	TAGCAGGCTCTTATTTTTCTTTTTG	GATGATCTTGATGCCTTGACCT
15	CAATGCTGAACAAATAAGG	AGCATTCCTTCTCCTTAACC
16	ATTCAATGCTGACACAAATAAGGTT	TTATCCCCAAGATGGCCTCAAA
17	TTTCTACTGTTTTCTTTGTCTGATA	GATCCTTGGGCTATAGACTGAA
18	TGACTTTTAAATTGCCACTGTCAAT	GACTTTATTTGGGTCATGTACCTT
19	ATCTGGGTGTACAACCTTGAAGT	TCTCGCAACATTATCATTTCCATTT
20	AGTGGTAGAAAAGAGGTTTCTGT	CCTGGGTAACAGAGTGAGACT
20	TGTAATTCAAAATGAACAGTAAAAATGA	GAAAGAAAGAAAGAAAGAAAGAAAGAAA
21	TAGACTTTCAAACTGAGCTCAGTA	TTTCATAATTACCCTTATCTTTCCAA
22	TCTCAATCATTCTGTGACATTTCA	GAGCAAAAACAAAAAAGTAGATTATTT
23	GTCAAAAGTATCCTTTGATTGGAAA	CTTCACCCCGCCCCCATATT
24	ATGATTAGACGGGCACTGTTAGA	ATTTGAGATTAAACTTGATTTGAAAGT
25	TACCTTTGCCTGATTTTTGACACA	TGAGCCATTCTCACAACTTCCAA
26	TACATAGCATCATAAATTTGTGACAT	GCATAAACAAACCTGCCAACTG
27	GCCATCAGTTTGACATGAGCATA	CCCAAACAATTGCATCTGCACAT

### Isolation of RNA

Total RNA was isolated from normal and RB retinal specimens, separately using RNeasy Mini kits (Qiagen, Valencia, CA). RNA quality and quantity were assessed using the Agilent Bioanalyzer 2100 (Agilent Life Sciences and Chemical Analysis, Santa Clara, CA). The yield of intact RNA from the RB tumors or matched retina was one of the limiting factors for gene expression analysis. Therefore, approximately 50 ng of total RNA was subjected to in vitro transcription yielding cRNA with the Ovation® RNA Amplification System V2 (NuGEN Technologies, San Carlos, CA) and the FL-Ovation cDNA Biotin Module V2 (NuGen Technologies), exactly according to the manufacturer's instructions. Approximately 40 µg cDNA was obtained and used in hybridization to microarrays.

### Microarray analysis of cDNA

cDNA was fragmented using DNASe1 to lengths of approximately 200 nucleotides, heated at 99 °C for 5 min, and hybridized for 16 h at 45 °C to the GeneChip® Human U133 V2.0 microarray (Affymetrix, Santa Clara, CA). The array set was washed at low stringency (6× SSPE) and high stringency (100 mM MES, 0.1 M NaCl) and stained with streptavidin–phycoerythrin (Affymetrix). Fluorescent signals were amplified by adding anti-streptavidin and an additional aliquot of streptavidin–phycoerythrin stain. A confocal scanner was used to collect the fluorescence signal at 3 mm resolution after excitation at 570 nm.

The array images were assessed by naked eye to confirm scanner alignment and the absence of significant bubbles or scratches. The ratios of 3′:5′ ends were assessed for glyceraldehyde-3-phosphate dehydrogenase and β-actin and were found to be within acceptable limits (1.39 to <10.0). When the average intensity of all genes on each array was compared to that of a target gene, using Affymetrix MAS 5.0 array analysis software, scaling factors for all arrays and background were within acceptable limits. The raw gene expression data were processed using the Affymetrix Gene Expression Console software.

### Microarray data analysis

Affymetrix expression analysis is known to be dependent on preprocessing and signal summarization protocols [16]. Therefore both the Affymetrix standard protocols and the standard model-based methods of robust multichip average were used. The robust multichip average is a summary measure of probes on arrays. The values are background adjusted, normalized, and log transformed.

### Significance analysis of microarrays

We have used the significance analysis of microarrays method for two-class unpaired data analysis to identify differentially expressed gene tags. The differentially expressed genes were identified, with a false discovery rate of 5% [17].

### Pathway analysis

After selection of the gene tags with significant differential expression, the list of gene tags and the fold changes were imported into the Ingenuity Pathways Analysis application (Redwood City, CA). This software identified the gene associated with each gene tag, the biologic functions, and the pathways most relevant to the genes of interest.

### Quantitative PCR analysis

Observed differences in the expression of selected genes were validated by Taqman®-based quantitative PCR analysis using the assays-on-demand service and the ABI Step-one Sequence Detection System (Applied Biosystems, Foster City, CA).

## Results

### Patients and clinical samples

Tumor and normal tissue were dissected from enucleated ocular material of six individuals with RB. There was no presurgical ocular therapy. It is rare to have matched normal retina and retinoblastoma tissue from the same individual because current recommendations for RB disease management avoid enucleation except in the severest cases. The six individuals in this study ranged in age from 9 months to 5 years. The clinical features were similar for all six cases. Five tumors were classified as stage E and one as stage D [18]. Two individuals with bilateral RB carried germline mutations (Table 1). The four individuals with unilateral disease did not carry any germline *RB1* mutations. Somatic mutations included loss of heterozygosity along with point mutations, rearrangements, and promoter methylation (Table 1). The adjacent normal tissue did not carry the somatic mutations identified in the matched RB.

### Gene expression analysis

We used the significance analysis of microarrays (SAM; SAM analysis method that ranks genes according to preselected level of FDR) [17] method for two-class unpaired data analysis to identify the differentially expressed gene tags with a false discovery rate which was set at 5%. Overall, out of total 33,326 tags, there were 1,116 individual expression tags that had elevated mean expression levels in tumor tissue compared to normal tissue (range 1.13 to 25 fold) and 837 tags that had reduced mean expression levels in tumor tissue compared to normal tissue (range −1.14 to −61.0 fold; Appendix 1).

To identify the functional classes represented by these gene tags, we performed a gene enrichment analysis using the Ingenuity Pathway Analysis software package. The molecular and cellular functions of both upregulated and downregulated genes with greatest representation in the gene list were cell cycle (n=309, p=1.86×10 ^−4^ to 4.6×10^−42^); cell death (n=437, p=1.7×10^−4^ to 3.24×10^−19^); DNA replication, recombination, and repair (n=270, p=1.83×10^−4^ to 4.84x10^−18^); cellular growth and proliferation (n=464, p=1.70×10 ^−4^ to 2.10^−17^); and cellular assembly and organization (n=110, p=1.83×10^−4^ to 1.11×10^−16^). The p values indicated a strong association between the number (n) of the molecules in the analyzed data set and the number of molecules in the reference data set for each function. The p value was calculated with the right-tailed Fisher’s exact test. The range was defined by the number of molecules in the data set that were associated with a certain function compared to the total number of molecules associated with that function. In addition, the total number of functional categories to which these molecules belong was also determined.

The top ten canonical pathways represented by the deregulated genes (Table 3) include the G_1_/S node of the cell cycle, the point at which the *RB1* gene is known to function. However, seven pathways involve DNA damage response and the G_2_/M node of the cell cycle. The pathways that received the most support were: DNA damage response and *BRCA1* gene, aryl hydrocarbon receptor (AHR) signaling, ATM signaling, G_2_/M DNA damage checkpoint regulation, mitotic roles of polo-like kinase (PLK), role of CHK proteins in cell-cycle checkpoint control, purine metabolism, molecular mechanisms of cancer, G_1_/S checkpoint regulation, and P53 signaling (Table 3). Thus, this data set reveals that multiple pathways are strongly affected in retinoblastoma tumors with p values ranging from 10^−12^ to 10^−2^ (column 2 of Table 3 indicates the negative log of the p values). Because DNA damage response is linked to DNA replication and proliferation, it is important to note that the expression of the KI67 marker was fourfold higher in RB compared to normal retina. The matched tissue in each case was adjacent normal retina from young children for whom the retinal was still maturing and changing [19]. These data are supported by similar observations in CRX-, RXR-γ-, and TRβ-2-positive proliferating cone cells in human RB [20].

**Table 3:** Canonical Pathways deregulated in RB tumor tissue.

**Canonical pathways**	**-Log (P-value)**	**Ratio of molecules in this data set compared to total number of molecules in reference**	**Down regulated molecules (compared to total number in the respective pathway)**	**Upregulated molecules (compared to total number in the respective pathway)**	**No overlap with data set**	**Genes**
DNA damage response and *BRCA1* gene	12	0.472	1/53 (2%)	24/53 (45%)	28/53 (53%)	*BARD1, RBBP8, E2F3, RBL1, SMARCA4, CHEK1, FANCE, RAD51, FANCD2, BRCA1, BLM, E2F2, RBL2, FANCG, PLK1, RPA1, RFC5, MSH2, RFC4, E2F1, MSH6, BRCA2, HLTF, FANCA, RFC3*
Aryl hydrocarbon receptor signaling	6.85	0.217	18/157 (11%)	16/157 (10%)	123/157 (78%)	*TRIP11, NFIX, GSTM5, TP73, POLA1, SMARCA4, CHEK1, TGM2, CCNA2, PTGES3 (includes EG: 10728), CCNA1, ALDH1A3, TGFB2, NFE2L2, ALDH6A1, GSTK1, CCNE2, NFIC, TFDP1, GSTM, NQO1, MAPK8, MDM2, NCOA3, RXRG, CCNE1, CCND2, NFIA, E2F1, ALDH3B1, DHFR, GSTO2, ESR1, MCM7*
ATM signaling	5.93	0.346	0/52 (0%)	18/52 (35%)	34/52 (65%)	*SMC3, CDC25C, CCNB1, TP73, MAPK8, MDC1, CCNB2, MDM2, CREB3L4, CDC2, SMC1A, CHEK1, RAD51, SMC2, FANCD2, TLK2, BRCA1, CDC25A*
G2/M DNA damage checkpoint regulation	5.93	0.349	2/43 (5%)	13/43 (30%)	28/43 (65%)	*PRKDC, CDC25C, CCNB1, YWHAB, WEE1, CCNB2, MDM2, PLK1, SKP1, CDC2, SKP2, CHEK1, KAT2B, TOP2A, BRCA1*
Mitotic roles of polo-like kinase	5.93	0.306	1/62 (2%)	18/62 (29%)	43/62 (69%)	*KIF23, CDC25C, CCNB1, ESPL1, CDC20, WEE1, PTTG1, PRC1, CDC7, CCNB2, PLK1, CDC23, CDC2, PPP2R5A, SMC1A, PLK4, PKMYT1, KIF11, CDC25A*
Role of CHK proteins in cell cycle checkpoint control	5.86	0.412	0/34 (0%)	14/34 (41%)	20/34 (59%)	*CDC25C, RPA1, RFC5, E2F3, CDC2, CHEK1, PCNA, RFC4, E2F1, TLK2, BRCA1, E2F2, RFC3, CDC25A*
Purine metabolism	5.31	0.125	12/440 (3%)	43/440 (10%)	385/440 (88%)	*PRIM1, POLR2D, PRTFDC1, POLA1, ADARB1, POLE, POLD3, PDE7B, PRUNE, RAD51C, AK7, GUCY1B3, GUCY1A3, POLE2, RRM2, ENPP5, PAPSS2, NT5C3, MPP3, POLD2, ATP6V0E1, PPAT, RFC3, DDX39, PDE7A, POLQ, PDE4A, POLR2B, SMARCA4, WRNIP1, RAD51, ENTPD3, ENPP2, BLM, DHX15, ADCY2, DCK, ENTPD1, PAICS, CASK, HSPD1, RFC*
Molecular Mechanisms of Cancer	4.64	0.156	27/372 (7%)	31/372 (8%)	314/372 (84%)	*TGFBR1, CDKN2C, RBL1, PMAIP1, NFKBIB, BRCA1, E2F2, CDC25A, CCNE2, TFDP1, RALB, RHOJ, AURKA, RASGRF2, DAXX, RHOQ, CCND2, RABIF, GAB1, ARHGEF6, E2F1, MAP2K3, PIK3CD, CFLAR, NOTCH1, TCF4, RALA, BMP2, CTNNA1, PSEN2, MAP3K5, E2F3, CHEK1, CASP6, FANCD2, RHOT1, GNAT2, TGFB2, PRKCE*
Cell Cycle: G1/S Checkpoint Regulation	3.68	0.254	4/59 (7%)	11/59 (19%)	44/59 (75%)	*CCNE2, RBL2, TFDP1, HDAC2, NRG1, RBL1, E2F3, SKP1, SKP2, CCNE1, CCND2, E2F1, TGFB2, E2F2, CDC25A*
P53 Signaling	3.47	0.225	5/89 (6%)	15/89 (17%)	69/89 (78%)	*PRKDC, PIK3C2A, TP73, MAPK8, MDM2, PTEN, BIRC5, CHEK1, CCNG1, KAT2B, CASP6, PCNA, CCND2, E2F1, BAI1, AKT3, PIK3CD, LRDD, BRCA1, PMAIP1*

* The p-value is defined as a measure of the likelihood that the association between the set of molecules differentially expressed in RB tumors and the given pathway is due to random chance. A smaller p-value indicates a statistically significant, non-random association.

### Presence of consensus sequences for binding to the E2F family of transcription factors

Historically, RB has been known to assert its role in cell-cycle progression through its interaction with members of the E2F family of transcription factors. To determine whether E2F-binding sites could be involved in the deregulation of selected genes in RB tumor tissue, we investigated the upstream promoter regions of the 20 most differentially expressed genes (Table 4). The consensus-binding sequence for the E2F family of transcription factors was represented by the motif M00050 [21]. Five of the ten most upregulated genes and two of the ten most downregulated genes contained the consensus-binding motif for E2F in their upstream promoter region (Table 4). Of all the genes that were expressed differentially in tumor versus normal retinal tissue, 47% of the promoters contained at least one E2F consensus sequence in the region spanning 5 kb of upstream genomic sequences of the respective genes (Appendix 2).

**Table 4:** Top 10 Molecules with highest and lowest fold change in expression between RB tumor and normal retina: molecules highlighted in bold contain the consensus sequence, M00050, in the 5-kb upstream region of the respective promoter for binding to E2F family of transcription factors.

**Upregulated genes**		**Down regulated genes**	
*NUF2*	25.229	***PDGFRA***	−61.726
***PBK***	23.8	*TYRP1*	−44.016
*CCNE2*	22.077	*ABCA8*	−39.006
*TOP2A*	22.031	*SCARA5*	−34.263
***C8ORF46***	21.409	*EGFL6*	−28.929
*SALL1*	20.017	*ENPP2*	−28.538
***RRM2***	19.616	*SYNPO2*	−22.238
***E2F7***	19.11	***CLIC5***	−21.56
*ASPM*	18.545	*GULP1*	−20.645
***CDCA7***	16.478	*PPAP2B*	−19.521

### Quantitative real-time PCR

Seven of ten non-overlapping canonical pathways included upregulation of CHK1—a checkpoint kinase that phosphorylates cell division cycle protein CDC25C. CDC25C is an important phosphatase in cell-cycle control, particularly for entry into mitosis. While an increase in CHK1 implies cell-cycle arrest, the increase in CDC25C has been documented in multiple cancers with poor prognosis [22].

**Figure 1:**
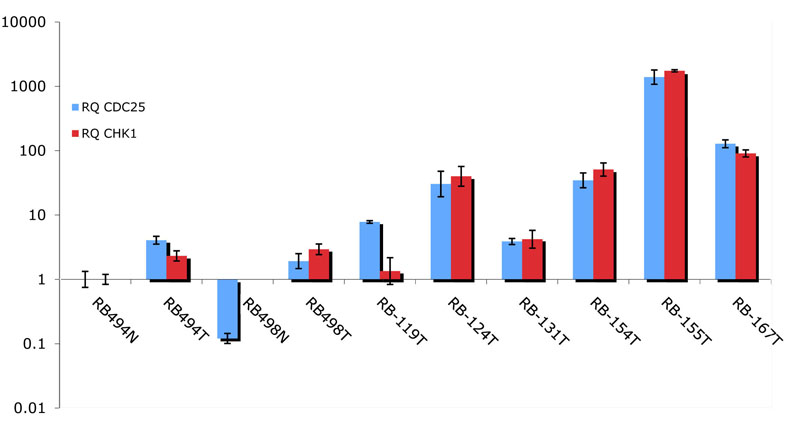
Relative degree of gene expression by qPCR. Validation of upregulation of gene expression for *CHK1* and *CDC25C* with qRT–PCR using independent normal retina (494N, 498N) and RB (494T, 498T, 119T, 124T, 131T, 154T, 155T, and 167T) samples. The relative quantity (RQ) value is compared to that of normal retina 494N. The error bars represent standard deviation from the mean.

The upregulation of *CHK1* and *CDC25C* genes was independently verified by quantitative real-time PCR on cDNA synthesized from two normal retina and eight tumor samples (Figure 1). All tumor samples showed elevated gene expression of *CHK1* as well *CDC25C* at the level of mRNA.

### *TP53* mutation analysis

It is known that pRB and p53 proteins work in parallel pathways to protect normal cells from DNA damage and replication errors. The coding exons 2 through 11 of the *TP53* gene were sequenced on tumor DNA, and no mutations were detected (data not shown). In addition, no alteration in *TP53* gene expression was observed between normal retina and retinoblastoma.

## Discussion

We have identified genes that are differentially expressed in retinoblastoma, using six matched sets of tumor and normal retinal tissues. The *RB1* gene was the first tumor suppressor gene to be identified and was defined as a negative regulator of the cell cycle [23–25]. Normally in its active form, pRB is hypo-phosphorylated and represses the transcription activity mediated by E2F1, and this leads to cell-cycle arrest at the G1/S junction blocking DNA replication in response to DNA damage. After damage is repaired, the pRB protein is phosphorylated and releases the E2F transcription factors, leading to activation or repression of the downstream targets. At the end of mitosis, the pRB protein is hypo-phosphorylated again and the cell cycle continues.

Since the transcription factor E2F1 is central to mediating the function of pRB, we expected that many of the genes that were found to be overexpressed or underexpressed in this study should have E2F1-binding sites in their promoter regions. We found this to be the case, with 47% of the cognate genes having a consensus E2F1-binding sequence. However, more than half of the deregulated genes had no E2F1-binding site. This is in agreement with other studies [21,26] showing that in vivo binding sites of the E2F family of genes often do not contain the consensus sequence or genes with consensus sequences in the promoter region are not in vivo targets of the same family of proteins. This implies multiple layers of protein–protein interactions involving pRB that can cause deregulation of genes in multiple pathways.

### Identification of multiple deregulated pathways in retinoblastoma tumorigenesis

Combining the data from six normal retina and RB showed that there were 1,116 individual gene tags that were elevated and 837 that were decreased. The genes identified by these tags were classified as being associated with five main molecular and cellular functions: cell cycle, cell death, DNA replication, recombination and repair, cellular growth and proliferation, and cellular assembly and organization.

**Figure 2:**
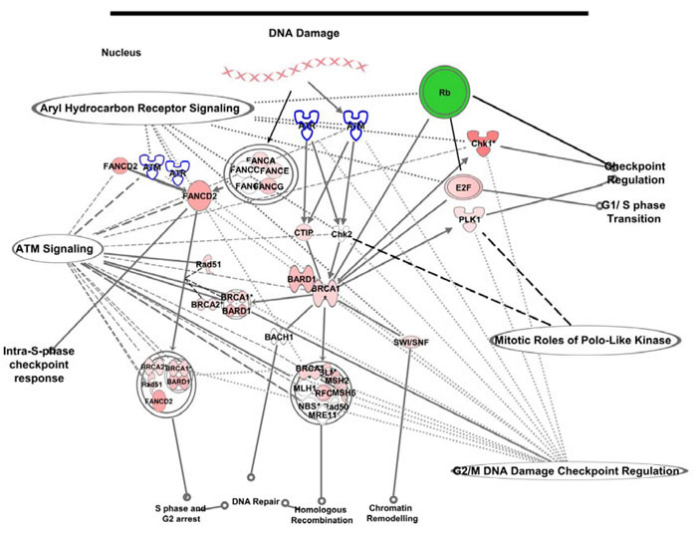
The concurrent deregulation of the top five canonical pathways in retinoblastoma (RB) tumors. The DNA damage response pathway with breast cancer associated gene 1 (*BRCA1*) at the center was the top canonical pathway identified with differential gene expression of the *RB1* gene. The other pathways were overlaid on this pathway retaining the relationships curated in the knowledge base of Ingenuity software (Redwood, CA).

The genes identified by their molecular functions belonged to multiple canonical pathways that were deregulated in the absence of normal functional pRB (Figure 2). This is represented by significantly more genes with altered expression than would be expected on the basis of chance alone (Table 3). Of the ten most significant canonical pathways, eight involved response to DNA damage. The pathway of G_1_/S DNA damage checkpoint was expected, but the pathways involving BRCA1 in DNA damage response, ATM signaling, and G_2_/M checkpoint regulation were not completely expected. In addition, the AHR signaling, modulation of mitotic cycle with PLK, and purine metabolism pathways have not been previously reported as associated with RB tumorigenesis.

The intricate network of these overlapping pathways is described in Figure 2. In response to *RB1* gene inactivation, indicated by the color green, different kinases and other molecules are upregulated and indicated by the red color of the molecules. Lines to the molecules connect the different affected pathways, indicated by their respective names. Multiple pathways share the same molecules.

The pathways identified in this study agree with the pathways observed to be altered by siRNA-mediated silencing of the *RB1* gene in human non-small cell carcinoma cells, H1299, in culture [27]. In that study, *RB1* gene knockdown affected G_1_/S and G_2_/M transitions of the cell cycle and showed significant involvement of the DNA damage response and repair pathways as well as epigenetic regulation of gene expression.

In another report comparing normal retina with RB samples, the PI3K/AKT/mTOR (insulin signaling) pathway was found to be aberrantly regulated [28]. This pathway was not identified as significantly altered in our data set. Next we compared the expression of *CDC25A*, *C17orf75*, *ERBB3*, *LATS2*, and *CHFR* between normal retina and RB. These genes were chosen because they were significantly altered in the other report [28]. We found that expression of *CDC25A* and *C17orf75* was upregulated in RB, while that of *LATS2* and *ERBB3* was downregulated (Appendix 1). However, there was no difference in expression of *CHFR* between normal retina and RB. This result can be partially explained by the fact that the ages of the individuals from whom normal retina was used in the previous study was 65 to approximately 80 years. In contrast, our study is based on data from matched normal retina belonging to children of ages 9 months to 5 years. As the retina continues to develop until the age of 5–10 years, the comparison of retinoblastoma with adult retina may lead to identification of alternate pathways.

The cell of origin for retinoblastoma has been elusive for many years. It was shown that for mouse retinoblastoma a horizontal interneuron was very likely to be the cell of origin [29,30]. In contrast for humans, retinoblastoma cone cells have been suggested as precursors for retinoblastoma [20,31]. In the latter publication, a very careful evaluation of the markers expressed in retinoblastoma was used to prove the cone cells as precursors of human retinoblastoma. With this background we compared the gene expression profile of the retinoblastoma tumors in the current data set for a subset of the genes expressing the marker proteins. Of three cone-specific markers, CRX, RXR-γ and TRβ−2, RXR-γ was expressed at an eightfold higher level in retinoblastoma compared to matched retina. The other two genes were not differentially expressed. In addition, *Pro × −1-*the progenitor cell-specific marker—was downregulated significantly but to a modest degree (−1.75 fold change). Finally, gene expression for both *N-MYC* (ninefold) and *MDM2* (1.9 fold) were upregulated in retinoblastoma compared to normal retina. Thus, the gene set identified in the current data set mimic what has been observed at the protein level in human retinoblastoma tumors. While it is interesting that many of these gene expression profiles matched the protein profiles of RB, it is possible that post-translational regulation of the mRNAs can explain the differential protein expression in RB tumors versus normal retina for respective genes with unchanged expression mRNA level.

AHR signaling is involved in cell proliferation and development. The AHR molecule is a cytosolic transcription factor that is usually inactive in the absence of ligands. However, exposure of cells to a wide variety of exogenous and endogenous ligands activates the AHR pathway that results in xenobiotic metabolizing enzymes. Interestingly, it has been demonstrated that pRB interacts with ligand-bound AHR and helps its translocation to the nucleus [32,33]. Additionally, recent studies have indicated that AHR is involved in the TGF- β1/SMAD pathway in glioblastoma pathogenesis [34]. It has also been suggested that AHR is a promising target of anticancer therapy and can modulate survival and invasiveness of malignant glioma [34].

Imbalance in the gene expression pattern of purine metabolism enzymes is linked with transformation and/or tumor progression [35]. In our data set, 12 genes in the purine metabolism pathway were downregulated, while 43 genes were upregulated (Table 3). A 16-gene expression signature of purine metabolism has been documented in acute lymphoblastic leukemia with or without the TEL–AML fusion gene product [36].

PLKs are a family of conserved serine/threonine kinases involved in the regulation of cell-cycle progression through G_2_ and M phases [37]. Inactivation of the RB pathway in cultured cells results in the deregulation of *PLK1* expression and can lead to errors in chromosome separation [38]. In addition, overexpression of *PLK1* in many cancers, including melanoma, ovarian cancer, and others, has been associated with poor prognosis [39]. The data set included in this report indicates that deregulation of *PLK* is also present in RB tumors. This observation is highly significant considering the large volume of literature on *PLK1* inhibitors and their ability to suppress tumor growth in vivo [40]. There are several ongoing clinical trials on other cancers using *PLK1* inhibitors; some of these may be potential therapeutic agents in RB as well.

Cell-cycle checkpoints ensure that DNA replication and mitosis occur only after all DNA damages are repaired or removed. In response to DNA damage, damage sensors, like *ATM* and *ATR* genes, are activated, and the resulting proteins in turn activate downstream molecules, such as the *BRCA1* gene product. BRCA1 can lead to activation of CHK1—a checkpoint protein that belongs to a family of serine-threonine kinases (Figure 2). Thus, it appears that the ATM–ATR–BRCA1–CHK1 axis in the DNA damage response pathway is one of the affected axes in RB (Figure 2).

The *CHK1* gene is involved in seven canonical pathways: DNA damage response and *BRCA1* gene, AHR signaling, ATM signaling, G_2_/M DNA damage checkpoint, role of CHK proteins in cell-cycle checkpoint, molecular mechanisms of cancer, and P53 signaling. The CHK protein is required for checkpoint-mediated cell-cycle arrest in response to DNA damage and is found in association with unreplicated DNA. ATM and ATR phosphorylate the CHK1 protein, and this results in enhanced CHK1 kinase activity. CDC25C is a protein phosphatase and a direct target of CHK1. In the absence of CHK1, CDC25C will dephosphorylate CDC2 and allow the cells to proceed through mitosis. As the activity of CHK1 increases, CDC25C is phosphorylated, which enhances binding to mitotic inhibitor proteins, such as 14–3–3. As a consequence G2/M progression is halted. Further studies are required to determine the levels of proteins and the phosphorylation status of the proteins expressed by the differentially expressed genes identified in this study.

The enhanced expression of CHK1 in RB suggests that tumor cells should be arrested and all damage repaired. However, in the absence of functional pRB protein, the cells proceed with mitosis and DNA replication. Following replication of damaged DNA, the tumor cells acquire many mutations and should eventually die. It is interesting to note that multiple genes with the “cell death” molecular function (Table 3) were differentially expressed. As the tumors are not regressed, it indicates that the cell death or apoptosis-related pathway is compromised in these retinoblastoma tumors.

The p53 protein, encoded by the *TP53* gene, is one of the gatekeepers of cell division and proliferation and is mutated or lost in a many cancers. We found that this gene was not differentially expressed in the retinoblastoma tissue. In addition, there was no mutation in the coding sequences of this gene, thus contraindicating a direct role of TP53-mediated apoptosis in retinoblastoma tumors.

In conclusion, our report partially defines the network of canonical pathways that are deregulated and that distinguish an RB tumor from adjacent normal retina. The differentially expressed genes are known to function in multiple pathways and thus suggest that pRB has multiple roles in cell-cycle control. It is known that pRB regulates the G_1_/S checkpoint, but this analysis highlights the role of pRB in the G_2_/M DNA damage checkpoint through novel pathways, including AHR signaling, PLK-mediated mitotic assembly, or purine metabolism pathways. This result was not unexpected since phosphorylated pRB releases the bound E2F family of transcription factors and the many downstream targets of E2F can modulate multiple pathways. In addition, pRB can modulate chromatin structure independent of E2F-binding activities [41], and thus pRB may deregulate genes without an E2F-binding site in their respective promoters. The most important observation is identification of novel pathways deregulated in RB. Currently several drugs are available for therapeutic targeting of the AHR-signaling pathway and the CHK1- and PLK-associated pathways. These drugs are being used in clinical trials for treatment of other cancers and should be investigated for treatment of RB. Many of these differentially expressed genes belong to proliferation pathways. A review article indicated that “the proliferation signature of tumor cells is comprised of cell cycle regulated genes whose pattern of expression is altered because a tumor contains more proliferating cells than the normal” [42]. However, the “proliferation signatures” can be different between different cancers. Therefore, in this manuscript we have defined the “proliferation gene signature of retinoblastoma” and aims to identify drugs that can alter this signature in favor of regression and cure.

## Appendix 1. List of genes with significant differential expression between retinoblastoma matched with normal retina.

To access the data, click or select the words “Appendix 1.” This will initiate the download of a compressed (pdf) archive that contains the file.

## Appendix 2. List of genes with consensus binding site, M00050, for E2F family of transcription factors.

To access the data, click or select the words “Appendix 2.” This will initiate the download of a compressed (pdf) archive that contains the file.
